# Comparable Effects of Brief Resistance Exercise and Isotime Sprint Interval Exercise on Glucose Homeostasis in Men

**DOI:** 10.1155/2017/8083738

**Published:** 2017-02-28

**Authors:** Tomas K. Tong, Zhaowei Kong, Xueying Shi, Qingde Shi

**Affiliations:** ^1^Dr. Stephen Hui Research Centre for Physical Recreation and Wellness, Department of Physical Education, Hong Kong Baptist University, Hong Kong; ^2^Faculty of Education, University of Macau, Macau; ^3^School of Physical Education and Sports, Macao Polytechnic Institute, Macau

## Abstract

This study compared the effects of a single bout of resistance exercise (RES) on glycemic homeostasis to isotime sprint interval exercise (SIE) using a within-subjects design. Nineteen nondiabetic males (age: 23.3 ± 0.7 yrs; height: 173.1 ± 1.2 cm; weight: 79.1 ± 4.8 kg; % fat: 22.5 ± 2.5%) were studied. RES involved nine exercises of 10 repetitions at 75% 1-RM using a 2 : 2 s tempo and was interspersed with a one-minute recovery; SIE involved four 30 s' all-out cycling effort interspersed with four minutes of active recovery. Plasma glucose and insulin in response to a 75 g oral glucose tolerance test were assessed 12 h after exercise. In comparison to a no exercise control trial (CON), the area under curve (AUC) of plasma glucose was reduced with both RES and SIE (*P* < 0.05), while insulin AUC was only reduced with RES. Cederholm, Gutt, Matsuda, and HOMA indices were improved (*P* < 0.05) following RES compared to CON. Corresponding changes following SIE were only found in Cederholm and Gutt indices (*P* < 0.05). No difference was found in plasma variables and indices between RES and SIE (*P* > 0.05). Such findings suggest that the RES may represent a potential alternative to the SIE in the development of time-efficient lifestyle intervention strategies for improving diabetes risk factors in healthy populations.

## 1. Introduction

Previous evidence suggests that an interval training regime consisting of short-term repeated cycling sprints could induce desired metabolic adaptations including increased insulin sensitivity, which are well known to be associated with traditional high-volume endurance training [1–4]. The relatively low volume of exercise in the interval training regime compared to that of traditional endurance training favors the development of time-efficient lifestyle intervention strategies for improving diabetes risk factors [5]. However, the feasibility of the sprint power profile which requires a specialized Wingate type cycle ergometer is still questionable to accomplish outside the laboratory setting [6]. Moreover, the outcomes of the significant metabolic benefits resulting from the repeated all-out exercise with considerable verbal encouragement are not guaranteed when the exercise is performed in a leisure environment.

Resistance exercise training has been hypothesized to reduce multiple health risk factors including those for cardiovascular disease [7]. In contrast to the sprint cycling protocol, a typical resistance workout, which consists of eight to ten exercises covering the major muscles of the trunk, arms, and legs, distributes the workload to different parts of the body instead of placing it on limited muscle groups. Fluckey et al. [8] have shown a significant reduction in serum insulin 18 h following a single session of resistance training composed of 3 sets × 10 repetitions in 7 exercises. However, the duration of the previous resistance exercise session was far beyond time-efficient in comparison to the 14 min intermittent cycling sprint protocol. Whether a bout of high-intensity resistance exercise, of brief duration, is comparable to that of the intermittent-sprint cycling and could act as a time-efficient strategy for improving glycemic homeostasis in normal adults has not been explored.

It is known that participation in exercise training on a long-term basis is associated with a lower prevalence of chronic diseases including cardiovascular disease and type 2 diabetes. Such favorable adaptations are likely to be a result of a cumulative acute exercise effect [9]. In fact, many of the nonstructural changes in the risk factors for cardiovascular disease and diabetes reported subsequent to exercise training were thought to be partly attributed to recent exertion [9]. It is logical to presume that a particular exercise training regime is effective in controlling chronic diseases when associated risk factors are reduced acutely with a single session of the training [10]. In this study, we examined the impact on the dynamics of glucose and insulin in response to an oral glucose tolerance test (OGTT) 12 h following a single bout of resistance exercise (RES) in nonobese healthy male adults, by comparing it with that resulting from a single bout of sprint interval exercise (SIE) and from no exercise [control (CON)]. The regime of the RES, which was modified from an exercise protocol previously applied to untrained persons [8], was composed of nine 40 s, 10-repetition-maximum (RM) exercises interspersed with a one-minute recovery. The total time commitment of 14 min for the RES was identical to that of SIE which consisted of four 30 s cycling sprints interspersed with a four-minute recovery. It was hypothesized that, in comparison to CON, both the RES and SIE could improve the glycemic homeostasis in the subjects, and the changes in the plasma glucose and insulin variables during the postexercise OGTT following the RES were comparable to those of SIE.

## 2. Methods 

### 2.1. Subjects

Thirty Chinese nondiabetic males participated in this study. Selection criteria included: (a) weight variation within 2 kg recorded in the previous six months, (b) free from hypertension, diabetes, and eating disorders, (c) nonsmokers, (d) not engaged in regular exercise, (e) no previous history of coronary heart disease or family history of early cardiac death (<40 years), and (f) requiring no long-term medication. Following an explanation of the purpose and constraints of the study and the potential benefits and risks involved in the exercises, subjects gave written informed consent for participation. The College Ethical Committee for the Use of Human and Animal Subjects in Research provided ethical approval for the study.

During experimental trials, ten of the thirty subjects were excluded due to a failure to fast overnight. One subject chose not to participate for personal reasons. Nineteen subjects (age: 23.3 ± 0.7 yrs; height: 173.1 ± 1.2 cm; weight: 79.1 ± 4.8 kg; % fat: 22.5 ± 2.5%) who completed all the experimental trials were investigated. The sample size of the present study was determined based on a previous study which examined the resistance training effect on insulin sensitivity of muscle and liver in twelve obese adolescents [11].

### 2.2. Preliminary Testing and Familiarization

Body height was measured to the nearest one millimeter using a wall-mounted stadiometer (Novel, Rockton, IL, US). Body weight (without shoes) and %fat were measured by the leg-to-leg bioimpedance measuring system (Inbody 720, Biospace, Seoul, Korea).

For the determination of 1-RM for each exercise in the RES, the procedure was described previously [12]. Briefly, subjects first warmed up with several sets of six to ten repetitions using a light load (40–60% estimated 1-RM). After that, subjects performed a single repetition with a load estimated at 90% of maximum. If the attempt succeeded, weight was added depending on the degree of effort applied to complete the former single repetition. If the attempt was unsuccessful, weight was reduced from the equipment. A minimum of five minutes of rest was given after each trial. The procedure was continued until failure to complete a single repetition through the full range of motion. The heaviest load completed properly was the 1-RM. The load of 10-RM applied in the RES was 75% of that of the 1-RM. Prior to the 1-RM measurement, the subjects' technique for completing each resistance exercise was approved by a certified personal trainer.

After the preliminary testing, familiarization trials with the 30 s Wingate exercise protocol and 10-RM resistance exercise protocol were undertaken to familiarize the subjects with the equipment, procedures, and the sensation of exercising to exhaustion.

### 2.3. Procedures

Following preliminary testing and familiarization, subjects completed three experiments on different days at the same time of day (8:00 p.m.): (1) sprint interval exercise (SIE), (2) resistance exercise (RES), and (3) remaining sedentary [control (CON)]. The three interventions were assigned in a random, balanced order and separated by one week. The interventions were ecologically valid as participation in physical activity in the evening is a common pattern in general young age populations [13]. Twelve hours following the exercise/nonexercise period in each trial, subjects underwent an OGTT by ingesting 75 g anhydrous glucose dissolved in 300 ml of water at around 9:00 a.m. Blood samples were collected before and 30, 60, 90, and 120 min after glucose intake (GI) to examine the changes in plasma glucose and insulin. One day prior to the OGTT in every trial, subjects were provided with the same meals (approximately 60% carbohydrate, 25% fat, 15% protein) at the same time and were instructed to prohibit food intake after 11:00 p.m. This allowed the subjects to fast for at least 10 hours before the OGTT. All trials were performed in an air-conditioned laboratory. Before each trial, the subjects refrained from participation in strenuous physical activity for at least one day.

### 2.4. Interventions

In the RES trial, subjects performed nine 10-RM exercises in the following sequence: left dumbbell lunge, two-arm dumbbell bent-over-row, right dumbbell lunge, dumbbell shoulder press, dumbbell chest press, dumbbell squat, abdominal crunch with dumbbell, dumbbell biceps curl, and dumbbell triceps extension. The sequence of the exercises complied with the ACSM guidelines for using large muscles followed by accessory muscles in resistance training [14]. Each bout of 10-RM exercise was performed for 40 s, with 60 s recovery in between two exercises. Each repetition of the exercises was completed within four seconds, with concentric and eccentric actions sharing two seconds in one repetition. A metronome set at 30 Hz was used for guidance. During the exercises, subjects were not given any verbal encouragement.

The SIE trial consisted of a 14 min sprint interval exercise period. The protocol characteristics comprised of four 30 s maximal exercise bouts interspersed with a four-minute active recovery. Subjects cycled maximally against a load equivalent to 7.5% body weight on a stationary cycle ergometer (Monark Ergomedic 839E, Monark, Sweden) during the 30 s exercise and cycled against a minimum load during the four-minute recovery. In each sprint, appropriate verbal encouragement was given to subjects by an experienced test administrator.

CON was a trial with conditions identical to those of the SIE and RES trials, but in place of exercising, subjects sat quietly for an equivalent period of time.

### 2.5. Measurements

In OGTT, with the subjects sitting, one milliliter venous blood from the antecubital vein was collected at the selected time points to examine the changes in plasma glucose, insulin, and C-peptide. Each blood sample collected was stored in EDTA tubes and kept on ice until the test terminated. The blood samples were then centrifuged (2500 rpm, 12 min) and plasma samples were collected afterward. All plasma samples were stored at −70°C and analyzed collectively after the completion of all experiments. As the recovery of the plasma volume change following resistance exercise and repeated-sprint exercise was reported to be complete within three hours [15, 16], no difference was assumed for the plasma volume in the OGTT of the three trials.

Plasma glucose was determined using the glucose oxidase method on a DXC-800 clinical system (Beckman Coulter, CA, USA). Plasma insulin was measured by the chemiluminescent enzyme immunoassay using the Immulite 1000 analyzer (Siemens Medical Solutions Diagnostics, CA, USA). Plasma C-peptide was determined by the electrochemiluminescence immunoassay using an automated immunoassay analyzer (Modular Analytics E170, Roche Diagnostics, Mannheim, Germany).

Plasma glucose and insulin values were plotted against corresponding time points. The area under the curve (AUC) of plasma variables for each trial was calculated, based on the conventional trapezoid rule, by applying GraphPad Prism 5.01 software (San Diego, CA, USA). Insulin sensitivity/resistance of subjects in each trial was revealed by calculating the insulin sensitivity/resistance indices through the equations shown in Table 1.

### 2.6. Data Analysis

The Kolmogorov-Smirnov normality test revealed that data for all variables were normally distributed. A two-way repeated measures ANOVA was used to examine the difference in the plasma variables in relation to the five time points and across the three trials. The differences in the insulin sensitivity and the AUC of the plasma variables between the three trials were examined using a one-way within-subjects ANOVA. Post hoc analyses using Newman-Keuls were performed when the main effect was significant. Effect sizes were calculated using Cohen's *d*. Relationships between variables were assessed using simple regression. All tests of statistical significance were assumed at a level of *P* < 0.05. All results are expressed as mean ± SEM.

## 3. Results

### 3.1. Exercise Measurements

All subjects were able to complete the four 30 s sprinting cycling bouts, and the nine 10-RM resistance exercises with maximum effort in the SIE and RES trials, respectively. The mean power outputs for the first to fourth cycling bouts of SIE were 449.0 ± 17.6 W, 381.0 ± 11.6 W, 301.8 ± 16.1 W, and 295.8 ± 13.0 W, respectively. The resistances applied during the exercises of RES were 38.4 ± 3.8 kg in left and right dumbbell lunges, 38.0 ± 4.8 kg in two-arm dumbbell bent-over-row, 25.7 ± 3.0 kg in dumbbell shoulder press, 33.0 ± 3.9 kg in dumbbell chest press, 41.1 ± 3.5 kg in dumbbell squat, 34.7 ± 3.9 kg in abdominal crunch with dumbbell, 22.0 ± 2.6 kg in dumbbell biceps curl, and 32.6 ± 4.1 kg in dumbbell triceps extension.

### 3.2. Plasma Variables

**Figure 1:**
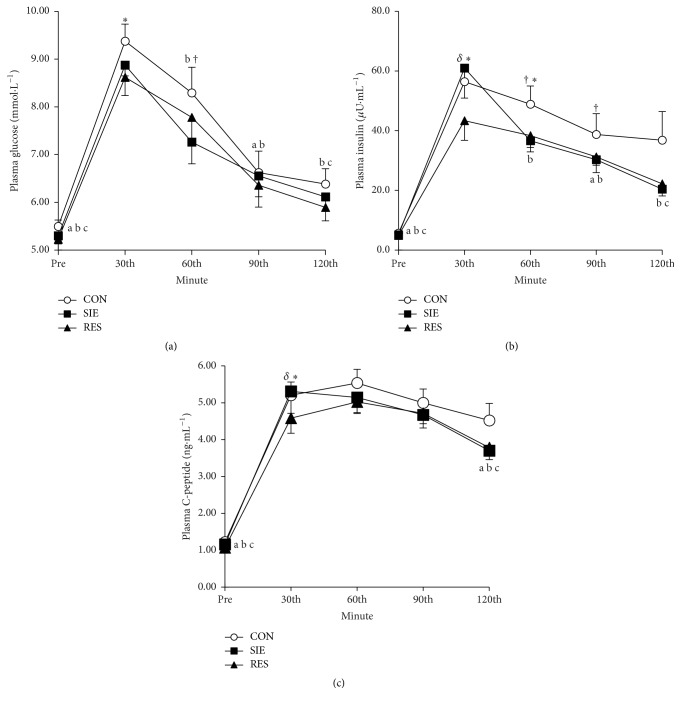
The time course changes in plasma (a) glucose, (b) insulin, and (c) C-peptide in response to 12 h postexercise OGTT in RES, SIE, and CON trials are shown. ^a^CON value significantly different from corresponding peak value; ^b^SIE value significantly different from corresponding peak value; ^c^RES value significantly different from corresponding peak value; ^*∗*^RES value significantly different from corresponding CON value; ^†^SIE value significantly different from corresponding CON value; ^*δ*^RES value significantly different from corresponding SIE value, *P* < 0.05.

The time course changes in plasma glucose, insulin, and C-peptide in response to the 12 h postexercise OGTT in RES, SIE, and CON trials are shown in Figure 1. The plasma glucose, insulin, and C-peptide in the three trials increased subsequent to the GI of the OGTT and decreased progressively following the time point of either 30 (glucose and insulin) or 60 min (C-peptide) (*P* < 0.05). In general, the increases in the plasma variables in the SIE and RES trials in response to the GI were relatively low in comparison to those of CON (*P* < 0.05). With the exception of the increase in the plasma insulin and C-peptide in the SIE trial at the 30-minute after GI which was higher than and close to, respectively, the corresponding values of RES and CON, the post-GI plasma variables were not different between SIE and RES trials (*P* > 0.05).

For the AUC of plasma glucose, SIE (852.0 ± 32.6 mmol·L^−1^·min) and RES (849.7 ± 41.2 mmol·L^−1^·min) were significantly less than those of CON (907.0 ± 40.1 mmol·L^−1^·min, *P* < 0.05). The difference in the AUC between SIE and RES was not significant (*P* > 0.05). For the plasma insulin, the AUC of the RES (3795.7 ± 343.6 mU·L^−1^·min) was significantly less than that of CON (4956.2 ± 567.6 mU·L^−1^·min, *P* < 0.05). The AUC of SIE (4219.2 ± 435.6 mU·L^−1^·min) did not differ significantly from that of CON and RES (*P* > 0.05).

### 3.3. Insulin Sensitivity/Resistance

Table 2 shows the simple surrogate indices for insulin sensitivity/resistance in the three trials. In comparison to CON, improved insulin sensitivity revealed in Cederholm (Cohen's* d*: 0.36) and Gutt (Cohen's* d*: 0.43) indices, but not Matsuda and IR_HOMA_ indices, was found with SIE (*P* < 0.05). Improved insulin sensitivity with RES was also found, manifesting not only in the indices of Cederholm (Cohen's* d*: 0.5) and Gutt (Cohen's* d*: 0.5), but also in that of Matsuda (Cohen's* d*: 0.6) and IR_HOMA_ (Cohen's* d*: −0.59). No difference was found between the indices of RES and SIE (*P* > 0.05). When the changes in the indices of the RES and SIE trials are expressed as a percentage of CON values, significant interindividual correlations in the changes were found in all indices (Cederholm *r* = 0.61, Gutt *r* = 0.65, Matsuda *r* = 0.67, IR_HOMA_  *r* = 0.71, *P* < 0.05).

## 4. Discussion

This study explored the potential of a brief resistance exercise protocol as an alternative to high-intensity intermittent cycling as a time-efficient intervention for attenuation of the risk of metabolic diseases. The present findings suggest that a brief single bout of either RES or SIE, with a total time commitment of 14 min, could acutely improve glycemic homeostasis in sedentary young adults. Indeed, when the subjects performed the brief, isotime SIE and RES, the glucose tolerance, in response to the 12-hour postexercise OGTT, improved in a similar magnitude from the corresponding CON values. Moreover, selected surrogate indices of peripheral insulin sensitivity (Cederholm and Gutt indices) derived from the OGTT results increased consistently following the two exercise bouts in comparison to those of CON. However, significant improvement in whole-body insulin sensitivity reflected by the Matsuda index was only found following RES, but not SIE. The sluggish response in the Matsuda index to the SIE was concomitant with an unchanged IR_HOMA_, suggesting that the underlying mechanism for the acute alterations in glucose and insulin dynamics in response to RES and SIE may not be identical. Nevertheless, the current findings of improved insulin sensitivity/resistance indices following RES imply the potential for the brief resistance exercise protocol in developing a time-efficient strategy for promoting glycemic homeostasis in normal adults.

**Figure 2:**
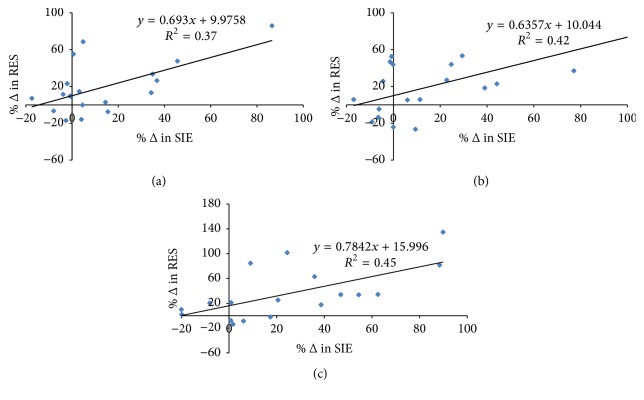
Interindividual correlation of the change (%  Δ) in the insulin sensitivity index of (a) Cederholm, (b) Gutt, and (c) Matsuda from corresponding CON value between RES and SIE (*r* = 0.61; *r* = 0.65; *r* = 0.67, resp., *P* < 0.05). Solid line is the line of regression.

Despite postexercise plasma glucose and insulin profiles and associated insulin sensitivity indices improving in general among the subjects, interindividual variability of the physiological response to exercise was noticeable. Among the nineteen subjects, significant reductions in the AUC of plasma glucose and insulin in response to the post-RES OGTT, in comparison to the CON values, were found in twelve and fifteen subjects, respectively. The rest of the subjects in contrast had the opposite results. The individual variations among the subjects in response to exercise were further evidenced by the significant correlations of the changes in the insulin sensitivity indices (*r* ≥ 0.61, *P* < 0.05) found between the SIE and RES trials (Figure 2). Such heterogeneous responses in the plasma variables among homogenous subjects are in accordance with the previous notions of interindividual differences existing in the outcomes of improvements in the intravenous glucose tolerance test following 20 weeks of cycling training [21] and 12 weeks of resistance training [11]. Genetic variation, training effort, and other unknown factors account for the varied individual responses [22].

Apart from the interindividual variability of the plasma glucose and insulin responses to exercise, the changes in the selected insulin sensitivity indices following RES and SIE were also inconsistent. The Matsuda index, which reflects a composite estimate of hepatic and peripheral (muscle) insulin sensitivity [23], only increased following RES but not SIE. The unchanged Matsuda index was concomitant with the unchanged IR_HOMA_ (decreased following RES) which has been used to assess hepatic insulin resistance more than peripheral insulin sensitivity based on fasting levels of glucose and insulin [24, 25]. In fact, the magnitude of the rise in the plasma glucose and insulin concentrations in the early phase of the OGTT (0–30 min), which is proportional to the magnitude of hepatic insulin resistance [26], was greater following SIE in comparison to RES (Figure 2). Such findings in conjunction with the increased indices of peripheral insulin sensitivity in the SIE trial suggest that the inconsistent change in the Matsuda index may be partly attributed to the different responses in hepatic, rather than, muscle insulin sensitivity to exercise. The present data could not explain clearly the possible existing discrepancy in the exercise-induced alterations in hepatic insulin resistance between RES and SIE. A recent study [27] demonstrated an increase in hepatic insulin resistance one hour following high-intensity exercise, while a similar change did not occur with moderate-intensity exercise. This transient tissue-specific insulin resistance may serve to spare carbohydrate for glycogen restoration in active muscles. Whether the relative high hepatic insulin resistance following SIE was a residual effect of the performance of high-intensity exercise is not known. It is also not known if such transient changes in hepatic insulin resistance have occurred following RES. Nevertheless, it appears that the hepatic insulin resistance revealed by IR_HOMA_ as well as the rise of plasma glucose and insulin in the early phase of OGTT is relatively less in RES compared with SIE. This is in agreement with recent findings of a reduction in IR_HOMA_ 24 hours following a circuit resistance exercise program [28]. Moreover, despite the difference in the increase in Cederholm and Gutt indices between RES and SIE not achieving statistical significance level, the increase in peripheral insulin sensitivity from the CON level tends to be higher following RES (Cohen's *d* effect size: Cederholm 0.5, Gutt 0.5) than SIE (0.36, 0.43). This may be due to the fact that the resistance exercise protocol compared with the cycling exercise involves muscle groups more comprehensively. In combining these two scenarios, a significant increase in the Matsuda index following RES resulted.

It is reasonable to postulate that the increased glucose tolerance as well as the insulin sensitivity after RES and SIE mainly originated in active muscles. However, the present findings are not able to reveal the precise underlying mechanism for the acute physiological responses following brief exercises. Long-term resistance training has been demonstrated to improve insulin sensitivity mainly in skeletal muscles [29]. Such training adaptation was partly attributed to the increase in glucose disposal resulting from augmentation of skeletal muscle mass [30]. Apparently, this should not be the case in the present study as muscle mass was unlikely to be augmented following a single bout of either SIE or RES. An increase in insulin clearance accompanied by unchanged glucose tolerance has been shown following resistance exercise [8], while further reports of supporting evidence for this are infrequent. In response to the postexercise OGTT in the present study, the time course of the plasma C-peptide, an indicator of *β*-cell activity of the pancreas, closely corresponded to that of plasma insulin, indicating that the reduction in insulin level and associated increase in insulin sensitivity should not be attributed to an increase in insulin clearance. There is a consensus that an increase in skeletal muscle glucose uptake during exercise in humans, including resistance exercise, is a result of enhanced glucose transporter 4 (GLUT-4) translocation [31, 32]. The activation of AMPK-p38 MAPK pathways in skeletal muscles is considered as one of the mechanisms underlying the enhancement of GLUT-4 during exercise, leading to increased glucose uptake [33, 34]. However, the increased p38 phosphorylation may not dominantly underlie the contraction-induced increase in muscle insulin sensitivity, as blocking the enzyme activity did not cease the physiological response [35]. Another postulation is that exercise could increase the rate of glucose uptake in the contracting skeletal muscles through insulin-independent mechanisms such as an increase in myoplasmic Ca^+2^ and AMP concentrations [31]. Such changes in the intracellular environment during contractions may activate the key signaling kinases of the Ca^+2^/calmodulin signaling pathway (CaMKII) and the AMPK-signaling pathway (AMPK), which may augment GLUT-4 translocation to the cell surface [36]. The great amount of GLUT-4 brought to the cell surface of skeletal muscles by contraction/exercise may enter into regions of high susceptibility to a weak insulin signal during endocytosis. As a result, the reversal of the increase in glucose transport after muscle contraction/exercise is replaced by a large increase in insulin sensitivity [37]. It was reported that a muscular contraction-induced increase in insulin sensitivity and associated glucose transport are mediated by a mechanism that may involve converging signaling between the insulin and the contraction pathway at downstream [31]; the precise assembly configuration of the mechanism, however, still awaits elaboration.

In conclusion, a single bout of RES, with a total time commitment of 14 min identical to that of repeating four 30 s cycling sprints interspersed with a four-minute recovery, appears to acutely improve glycemic homeostasis in nondiabetic male adults. The present findings provide further evidence for brief resistance exercise as a potential alternative to sprint interval exercise in the development of a time-efficient lifestyle intervention strategy for improving diabetes risk factors in nondiabetic healthy populations. Croymans et al. [22] have demonstrated the beneficial effects of 12 weeks of traditional resistance training on muscle insulin sensitivity and *β*-cell function in overweight/obese young men. Whether similar adaptations on insulin action can be obtained from a novel resistance training regime consisting of a brief exercise protocol in nondiabetic sedentary young adults awaits further investigation.

## Figures and Tables

**Table 1:** Indexes of insulin sensitivity/resistance derived from OGTT measurements of glucose and insulin.

Indexes	Equations
Cederholm [17]	$\frac{\left\{75000+\left[G_{0}\left(mg\cdot L^{-1}\right)-G_{120}\right]\times 0.19\times body\;\;weight\;\;\left(kg\right)\right\}}{\left\{120\times G_{mean}\left(mmol\cdot L^{-1}\right)\times log\left[I_{mean}\left(mU\cdot L^{-1}\right)\right]\right\}}$
Gutt [18]	$\frac{\left\{75000+\left[G_{0}\left(mg\cdot dL^{-1}\right)-G_{120}\right]\times 0.19\times body\;\;weight\;\;\left(kg\right)\right\}}{\left\{120\times log\left[I_{0,120mean}\left(mU\cdot L^{-1}\right)\right]\times G_{0,120mean}\left(mmol\cdot L^{-1}\right)\right\}}$
Matsuda [19]	$\frac{10000}{{\left[G_{0}\left(mg\cdot dL^{-1}\right)\times I_{0}\left(mU\cdot L^{-1}\right)\times G_{mean}\left(mg\cdot dL^{-1}\right)\times I_{mean}\left(mU\cdot L^{-1}\right)\right]}^{1/2}}$
IR_HOMA_ [20]	$\frac{\left[I_{0}\left(mU\cdot L^{-1}\right)\times G_{0}\left(mmol\cdot L^{-1}\right)\right]}{22.5}$

*Notes*. *G*_0_ is fasting plasma glucose concentration; *I*_0_ is fasting plasma insulin concentration; *G*_120_ is plasma glucose concentration in the 120th min of OGTT; *G*_mean_ is mean plasma glucose concentration during OGTT; *I*_mean_ is mean plasma insulin concentration during OGTT; *G*_0,120mean_ is average of *G*_0_ and *G*_120_; *I*_0,120mean_ is average of *I*_0_ and *I*_120_.

**Table 2:** Insulin sensitivity of subjects in the CON, SIE, and RES trials.

Indexes	CON	SIE	RES
Cederholm	51.4 ± 4.3	55.5 ± 3.6^*∗*^	61.7 ± 6.4^*∗*^
(mg·L^2^·mmol·L^−1^·mU^−1^·min^−1^)			
Gutt	82.5 ± 6.5	93.5 ± 7.2^*∗*^	100.3 ± 9.8^*∗*^
(mg·L^2^·mmol·L^−1^·mU^−1^·min^−1^)			
Matsuda	4.53 ± 0.71	4.91 ± 0.69	5.90 ± 1.04^*∗*^
IR_HOMA_	1.40 ± 0.13	1.20 ± 0.06	1.13 ± 0.07^*∗*^
(mmol·L^−1^·mU^−1^·L^−1^)			

Mean ± SEM

^*∗*^Significant different from corresponding CON value, *P* < 0.05.
